# Supplementary material for: Effects of Light on Growth and Metabolism of Rhodococcus erythropolis
Source: Microorganisms. 2022 Aug 20;10(8):1680. doi: 10.3390/microorganisms10081680 (PMC9416670; doi:10.3390/microorganisms10081680)
Supplement: Supplementary file 1 [file microorganisms-10-01680-s001.zip › Supplementals.pdf]

Effects of Light on Growth and Metabolism of *Rhodococcus erythropolis*

Supplementals

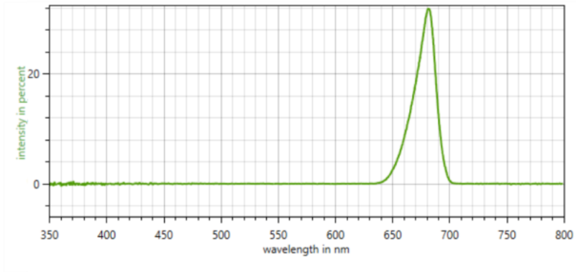

(**a**)

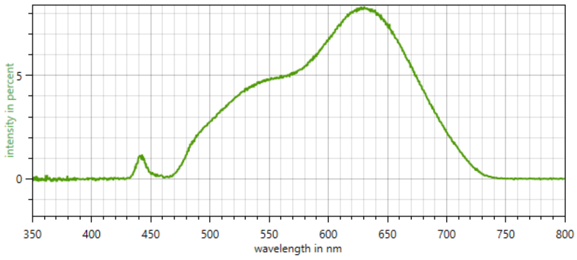

(**b**)

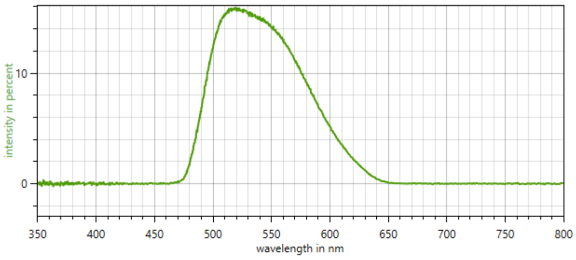

(**c**)

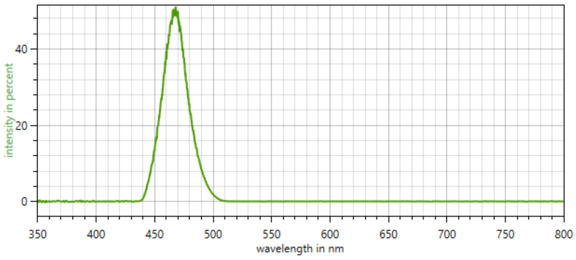

(**d**)

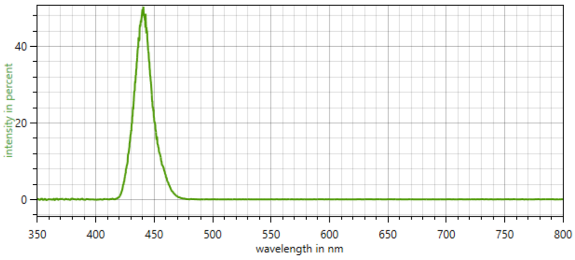

(**e**)

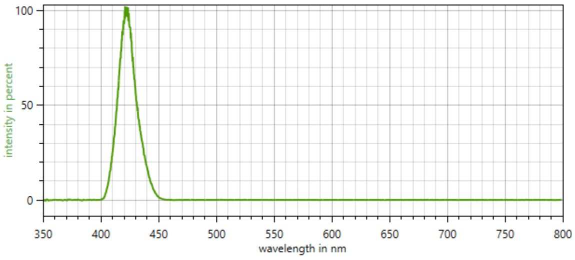

(**f**)

Figure S1: Spectrum of light (a) red light (680 nm) (b) white light (SWW) (c) green light (510 nm) (d) blue light (470 nm) (e) blue light (455 nm) (f) blue light (425 nm)

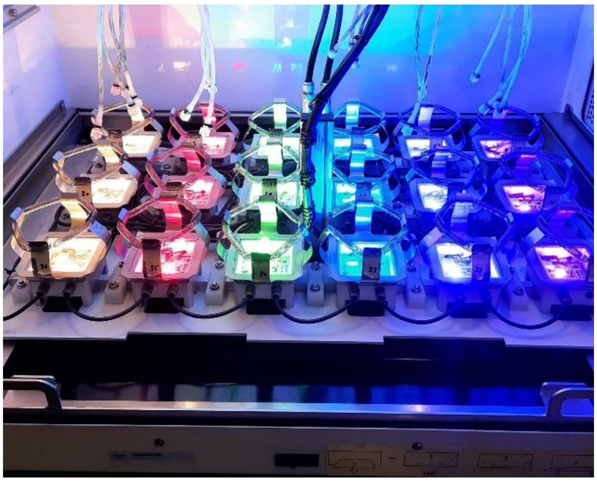

(**a**)

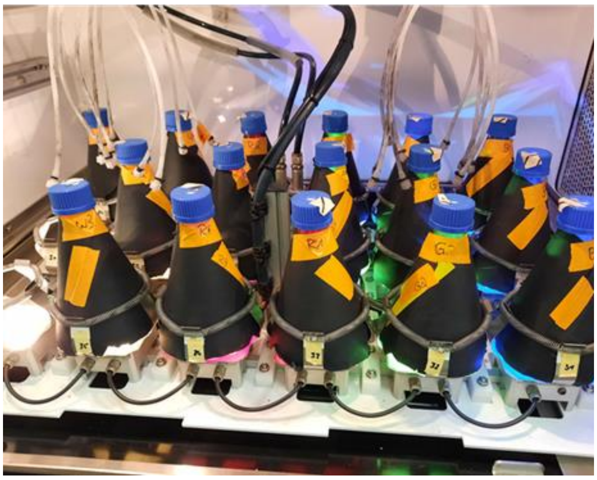

(**b**)

Figure S2. Customized shaking incubator (a)illumination setup with light of different wavelengths (b) shaded *R. erythropolis* cultures

Table S2. OD_{600nm} of *R. erythropolis* at 236 W s^{-2} irradiance with white, blue, green and red light illumination as well as dark conditions as control (D) (n=3).

Setup 1

	Time [h]	0	17	24	40	48,5	65	72	89	96	113	122
D	1	0,50	6,38	9,48	8,95	8,54	6,66	7,12	6,46	6,70	5,50	6,48
	2	0,50	6,45	9,92	8,35	8,12	6,90	6,98	6,14	6,18	5,52	6,52
	3	0,50	6,26	8,14	7,94	7,44	5,78	6,44	5,56	5,98	5,30	5,94
	Mean	0,50	6,36	9,18	8,42	8,03	6,45	6,85	6,05	6,29	5,44	6,31
	SD	0,0000	0,0961	0,9271	0,5070	0,5551	0,5897	0,3591	0,4562	0,3717	0,1217	0,3239
R	1	0,50	6,46	8,26	7,58	6,70	5,38	6,04	5,28	6,10	4,94	5,86
	2	0,50	6,00	8,80	8,38	8,62	7,66	7,40	6,92	6,64	6,40	7,04
	3	0,50	6,83	11,08	8,83	8,20	6,46	6,68	6,22	6,36	6,04	5,98
	Mean	0,50	6,43	9,38	8,26	7,84	6,50	6,71	6,14	6,37	5,79	6,29
	SD	0,0000	0,4158	1,4968	0,6315	1,0094	1,1405	0,6804	0,8229	0,2701	0,7606	0,6494
W	1	0,50	5,54	6,06	6,22	5,52	4,38	5,04	4,26	4,24	4,42	5,06
	2	0,50	5,68	6,20	5,62	5,32	4,80	5,58	4,62	4,30	4,26	4,98
	3	0,50	5,40	7,90	5,23	6,88	6,30	6,52	5,82	6,10	5,12	5,72
	Mean	0,50	5,54	6,72	5,69	5,91	5,16	5,71	4,90	4,88	4,60	5,25
	SD	0,0000	0,1400	1,0243	0,4959	0,8488	1,0094	0,7490	0,8168	1,0570	0,4574	0,4061
G	1	0,50	5,01	5,90	7,25	7,28	6,16	6,20	5,38	3,70	4,82	5,34
	2	0,50	5,55	6,96	6,26	5,98	4,68	5,14	4,84	3,42	3,92	4,86
	3	0,50	4,80	5,54	5,35	5,18	4,10	4,64	3,72	5,20	3,58	4,20
	Mean	0,50	5,12	6,13	6,29	6,15	4,98	5,33	4,65	4,11	4,11	4,80
	SD	0,0000	0,3875	0,7382	0,9482	1,0599	1,0623	0,7966	0,8467	0,9571	0,6407	0,5724
B455	1	0,50	3,27	4,70	4,82	4,80	3,66	4,40	3,74	2,78	3,78	4,12
	2	0,50	3,27	4,06	3,70	5,46	3,14	4,12	3,92	3,64	3,70	4,02
	3	0,50	3,54	4,74	4,85	4,78	3,82	4,08	3,48	4,88	3,68	4,10
	Mean	0,50	3,36	4,50	4,46	5,01	3,54	4,20	3,71	3,77	3,72	4,08
	SD	0,0000	0,1559	0,3816	0,6583	0,3870	0,3555	0,1744	0,2212	1,0557	0,0529	0,0529

Setup 2

	Time [h]	0	16	24	40	46	64	70	88	94
D	1	0,50	6,52	6,52	5,81	5,86	5,63	5,25	5,04	5,00
	2	0,50	6,43	6,62	5,95	6,10	5,44	5,37	5,08	4,96
	3	0,50	6,11	6,74	5,90	5,94	5,48	5,18	4,93	4,89
	Mean	0,50	6,35	6,63	5,89	5,97	5,52	5,27	5,02	4,95

	SD	0,0000	0,2155	0,1102	0,0709	0,1222	0,1002	0,0961	0,0777	0,0557
R	1	0,50	6,45	6,42	5,58	5,66	5,24	5,11	4,72	5,02
	2	0,50	6,37	6,55	5,57	5,96	5,27	5,20	4,56	4,95
	3	0,50	6,42	6,45	5,55	5,79	5,24	5,10	4,62	4,99
	Mean	0,50	6,41	6,47	5,57	5,80	5,25	5,14	4,63	4,99
	SD	0,0000	0,0404	0,0681	0,0153	0,1504	0,0173	0,0551	0,0808	0,0351
W	1	0,50	5,49	6,25	5,80	5,77	5,19	5,02	4,55	4,70
	2	0,50	5,61	6,36	5,54	5,66	5,24	4,93	4,47	4,75
	3	0,50	5,75	6,38	5,68	5,76	5,20	4,91	4,37	4,58
	Mean	0,50	5,62	6,33	5,67	5,73	5,21	4,95	4,46	4,68
	SD	0,0000	0,1301	0,0700	0,1301	0,0608	0,0265	0,0586	0,0902	0,0874
G	1	0,50	5,10	5,83	5,83	5,52	5,16	4,85	4,19	4,57
	2	0,50	5,38	5,93	5,57	5,56	5,00	4,90	4,29	4,70
	3	0,50	5,31	6,08	5,80	5,78	5,20	4,94	4,26	4,57
	Mean	0,50	5,26	5,95	5,73	5,62	5,12	4,90	4,25	4,61
	SD	0,0000	0,1457	0,1258	0,1422	0,1400	0,1058	0,0451	0,0513	0,0751
B470	1	0,50	4,28	4,85	5,22	5,22	4,73	4,80	3,91	4,30
	2	0,50	4,27	4,67	4,90	5,31	4,76	4,61	4,14	4,26
	3	0,50	4,23	4,91	5,25	5,40	5,08	4,64	4,23	4,40
	Mean	0,50	4,26	4,81	5,12	5,31	4,86	4,68	4,09	4,32
	SD	0,0000	0,0265	0,1249	0,1940	0,0900	0,1940	0,1021	0,1650	0,0721
B455	1	0,50	3,26	3,79	3,99	3,98	3,85	3,72	3,37	3,46
	2	0,50	3,06	3,40	4,40	4,16	3,93	3,76	3,51	3,61
	3	0,50	3,06	3,44	3,81	3,95	3,73	3,69	3,56	3,52
	Mean	0,50	3,13	3,54	4,07	4,03	3,84	3,72	3,48	3,53
	SD	0,0000	0,1155	0,2146	0,3024	0,1136	0,1007	0,0351	0,0985	0,0755
B425	1	0,50	2,60	3,04	3,38	3,70	3,52	3,27	3,45	3,38
	2	0,50	2,79	3,18	3,53	3,60	3,48	3,37	3,21	3,12
	3	0,50	2,63	3,05	3,15	3,68	3,34	3,56	3,37	3,24
	Mean	0,50	2,67	3,09	3,35	3,66	3,45	3,40	3,34	3,25
	SD	0,0000	0,1021	0,0781	0,1914	0,0529	0,0945	0,1473	0,1222	0,1301

Table S3. Dry cell weight (DCW) of *R. erythropolis* at 236 W s^{-2} irradiance with white, blue, green and red light illumination as well as dark conditions as control (D) (n=3)

Setup 1

	**40 h**						
	Empty [g]	Empty + Pellet [g]	Pellet only [g]	g/L	Mean	SD	T-test
**D**	12,7007	12,7466	0,0459	1,8360	1,7960	0,0799	
	12,8596	12,9058	0,0462	1,8480			
	12,8159	12,8585	0,0426	1,7040			

**R**	12,7178	12,7593	0,0415	1,6600	1,7973	0,1191	0,98792272
	12,6015	12,6483	0,0468	1,8720			
	12,8880	12,9345	0,0465	1,8600			
**W**	12,7287	12,7641	0,0354	1,4160	1,6173	0,3015	0,37731661
	12,8112	12,8480	0,0368	1,4720			
	12,7894	12,8385	0,0491	1,9640			
**G**	12,8130	12,8528	0,0398	1,5920	1,4187	0,1701	0,02540509
	12,8142	12,8495	0,0353	1,4120			
	12,8465	12,8778	0,0313	1,2520			
**455**	12,8446	12,8639	0,0193	0,7720	1,0427	0,2783	0,01076795
	12,8833	12,9165	0,0332	1,3280			
	12,8442	12,8699	0,0257	1,0280			

	**122 h**						
	Empty [g]	Empty + Pellet [g]	Pellet only [g]	g/L	Mean	SD	T-test
**D**	12,8383	12,8884	0,0501	2,0040	1,996	0,0883	
	12,8599	12,9075	0,0476	1,9040			
	12,6842	12,7362	0,0520	2,0800			
**R**	12,5771	12,6224	0,0453	1,8120	2,096	0,2527	0,552840152
	12,9807	13,0352	0,0545	2,1800			
	12,9072	12,9646	0,0574	2,2960			
**W**	12,5792	12,6139	0,0347	1,3880	1,7067	0,3444	0,231451611
	12,7395	12,7810	0,0415	1,6600			
	12,7107	12,7625	0,0518	2,0720			
**G**	12,8406	12,8836	0,0430	1,7200	1,5480	0,2321	0,035362144
	12,5807	12,6217	0,0410	1,6400			
	12,6632	12,6953	0,0321	1,2840			
**455**	12,8255	12,8504	0,0249	0,9960	1,0213	0,0510	7,78355E-05
	12,8223	12,8493	0,0270	1,0800			
	12,8788	12,9035	0,0247	0,9880			

Setup 2

**40 h**

	Empty [g]	Empty + Pellet [g]	Pellet only [g]	g/L	Mean	SD	T-test
**D**	12,8550	12,9005	0,0455	1,8200	1,8013	0,0257	
	12,6895	12,7348	0,0453	1,8120			
	12,7066	12,7509	0,0443	1,7720			
**R**	12,8340	12,8788	0,0448	1,7920	1,7680	0,0262	0,19111057
	12,8588	12,9023	0,0435	1,7400			
	12,8473	12,8916	0,0443	1,7720			
**W**	12,6699	12,7147	0,0448	1,7920	1,7827	0,0349	0,49758532
	12,8908	12,9344	0,0436	1,7440			
	12,7791	12,8244	0,0453	1,8120			
**G**	12,8210	12,8681	0,0471	1,8840	1,8453	0,0335	0,14573179
	12,8181	12,8638	0,0457	1,8280			
	12,5686	12,6142	0,0456	1,8240			
**425**	12,8141	12,8473	0,0332	1,3280	1,2747	0,0855	0,00051804
	12,8712	12,9042	0,033	1,3200			
	12,8050	12,8344	0,0294	1,1760			
**455**	12,7994	12,8320	0,0326	1,3040	1,3520	0,0550	0,00021339
	12,8877	12,9230	0,0353	1,4120			
	12,8210	12,8545	0,0335	1,3400			
**470**	12,5756	12,6193	0,0437	1,7480	1,6760	0,0741	0,05041872
	12,6226	12,6626	0,0400	1,6000			
	12,5980	12,6400	0,0420	1,6800			

	**94 h**						
	Empty [g]	Empty + Pellet [g]	Pellet only [g]	g/L	Mean	SD	T-test
**D**	12,5978	12,6718	0,0740	1,6444	1,6563	0,0413	
	12,5894	12,6660	0,0766	1,7022			
	12,5980	12,6710	0,0730	1,6222			
**R**	12,8525	12,9266	0,0741	1,6467	1,6481	0,0013	0,749851349
	12,8202	12,8944	0,0742	1,6489			
	12,8335	12,9077	0,0742	1,6489			
**W**	12,8419	12,9114	0,0695	1,5444	1,5963	0,0449	0,163687851
	12,7139	12,7869	0,0730	1,6222			
	12,8086	12,8816	0,0730	1,6222			
**G**	12,5752	12,6436	0,0684	1,5200	1,5237	0,0084	0,005508964
	12,8405	12,9088	0,0683	1,5178			

	12,8670	12,9360	0,0690	1,5333			

**425**	12,8456	12,8945	0,0489	1,0867	1,0711	0,0214	2,62675E-05
	12,8749	12,9235	0,0486	1,0800			
	12,8964	12,9435	0,0471	1,0467			

**455**	12,8379	12,8837	0,0458	1,0178	1,0644	0,0404	5,93447E-05
	12,7062	12,7552	0,0490	1,0889			
	12,5879	12,6368	0,0489	1,0867			

**470**	12,8980	12,9641	0,0661	1,4689	1,4644	0,0044	0,001323559
	12,6905	12,7564	0,0659	1,4644			
	12,6994	12,7651	0,0657	1,4600			

Table S4. Carotenoid accumulation of *R. erythropolis* at 236 W s^{-2} irradiance with white, blue, green and red light illumination as well as dark conditions as control (D) (n=3)

Setup 1

	**40 h**					
	DCW [mg]	Abs_{454nm}	Abs_{454nm}/mg_{DCW}	Mean	SD	T-test
**D**	16	0,157	0,0098125	0,00965936	0,00056629	
	15,5	0,14	0,009032258			
	15	0,152	0,010133333			
**R**	15	0,139	0,009266667	0,00927083	0,00046043	0,408684
	16	0,141	0,0088125			
	15	0,146	0,009733333			
**W**	15	0,163	0,010866667	0,01219211	0,00115701	0,02713917
	15,5	0,197	0,012709677			
	15	0,195	0,013			
**G**	15	0,211	0,014066667	0,0145371	0,00041615	0,00027445
	16	0,235	0,0146875			
	14	0,208	0,014857143			
**455**	15,5	0,113	0,007290323	0,00809677	0,00081942	0,05314016
	14	0,125	0,008928571			
	14	0,113	0,008071429			

**122 h**

	DCW [mg]	Abs_{454nm}	Abs_{454nm}/mg_{DCW}	Mean	SD	T-test
**D**	15	0,182	0,012133333	0,01273799	0,00069679	
	15,5	0,195	0,012580645			
	14	0,189	0,0135			
**R**	15	0,149	0,009933333	0,01079444	0,0012275	0,07558031
	15	0,183	0,0122			
	16	0,164	0,01025			
**W**	15	0,178	0,011866667	0,01170046	0,00054011	0,11114665
	15,5	0,172	0,011096774			
	14,5	0,176	0,012137931			
**G**	15	0,195	0,013	0,01164887	0,00249103	0,50623322
	15,5	0,136	0,008774194			
	14,5	0,191	0,013172414			
**455**	14,5	0,077	0,005310345	0,00539751	0,00048153	0,00011475
	14,5	0,072	0,004965517			
	12	0,071	0,005916667			

Setup 2

	**40 h**					
	DCW [mg]	Abs_{454nm}	Abs_{454nm}/mg_{DCW}	Mean	SD	T-test
**D**	15,1	0,128	0,008476821	0,00845661	0,00021194	-
	14,9	0,129	0,008657718			
	15,3	0,126	0,008235294			
**R**	14	0,111	0,007928571	0,00853164	0,00066528	0,86141635
	15,9	0,147	0,009245283			
	15,2	0,128	0,008421053			
**W**	14,1	0,137	0,009716312	0,01086662	0,00120471	0,02696424
	15,1	0,183	0,012119205			
	15,7	0,169	0,010764331			
**G**	13,9	0,171	0,012302158	0,01298628	0,0010712	0,00198779
	15,4	0,219	0,014220779			
	15,6	0,194	0,012435897			
**425**	14,2	0,142	0,01	0,00928114	0,00093641	0,21109544

	13,2	0,127	0,009621212			
	13,5	0,111	0,008222222			

**455**	15,2	0,103	0,006776316	0,00704363	0,00034012	0,00363883
	13,6	0,101	0,007426471			
	15,3	0,106	0,006928105			

**470**	14,3	0,216	0,015104895	0,01596651	0,00087648	0,00013425
	14	0,236	0,016857143			
	16	0,255	0,0159375			

	**94 h**					
	DCW [mg]	Abs_{454nm}	Abs_{454nm}/mg_{DCW}	Mean	SD	T-test
**D**	15,1	0,112	0,007417219	0,00742067	0,00046877	-
	14,7	0,116	0,007891156			
	15,1	0,105	0,006953642			
**R**	15,2	0,115	0,007565789	0,00765663	0,00020128	0,46795091
	14,2	0,112	0,007887324			
	14,9	0,112	0,007516779			
**W**	14,3	0,158	0,011048951	0,01113713	0,00052504	0,00079337
	13,6	0,145	0,010661765			
	14,7	0,172	0,01170068			
**G**	15,9	0,16	0,010062893	0,01003411	0,00053642	0,00314346
	14,4	0,152	0,010555556			
	15,5	0,147	0,009483871			
**425**	15	0,063	0,0042	0,00445458	0,00030233	0,00077216
	14,2	0,068	0,004788732			
	16	0,07	0,004375			
**455**	14,5	0,061	0,004206897	0,00399575	0,00019462	0,00030645
	13,6	0,052	0,003823529			
	13,9	0,055	0,003956835			
**470**	14,2	0,143	0,010070423	0,01072982	0,00063331	0,00189736
	15	0,17	0,011333333			
	14	0,151	0,010785714			

Table S5. Fatty acid profile of *R. erythropolis* at 236 W s^{-2} irradiance with white, blue, green and red light illumination as well as dark conditions as control (n=3) in µg mg^{-1}_{DCW}.

Setup 1

	40 h				
Fatty acid	Dark	Red680	WhiteSWW	Green510	Blue455
C13:0	n.d.	0.06±0.1	0.43±0.03	0.22±0.05	n.d.
C14:0	4.34±0.04	4.54±0.11	3.82±0.1	3.59±0.16	3.27±0.13
C14:1	0.66±0.06	0.63±0.14	0.37±0.03	0.41±0.06	0.21±0.2
C15:0	1.52±0.04	1.52±0.02	3.53±0.04	4.15±0.2	0.78±0.07
C16:0	9.24±0.13	9.9±0.18	8.24±0.18	8.18±0.13	12.27±0.34
C16:1	9.46±0.06	9.15±0.45	6.75±0.08	6.62±0.18	5.98±0.35
C17:00	0.38±0.03	0.41±0.03	0.7±0.05	0.96±0.13	0.64±0.05
C17:1	2.1±0.04	2.04±0.05	2.68±0.12	2.86±0.13	0.97±0.05
C18:0	0.24±0.08	0.17±0.17	0.05±0.09	0.68±0.8	0.32±0.1
C18:1 (oleat)	2±0.05	2.02±0.04	0.77±0.01	0.8±0.11	1.46±0.08
C18:1 (vaccenat)	17.04±0.12	16.98±0.27	13.62±0.19	13.83±0.37	15.33±0.1
C18:2	n.d.	n.d.	n.d.	n.d.	n.d.
C18:3	2.33±0.03	2.46±0.04	2.72±0.12	2.89±0.25	1.59±0.12
C19:00	n.d.	n.d.	n.d.	0.18±0.31	n.d.
C20:0	n.d.	n.d.	0.25±0.03	0.27±0.03	n.d.
C20:1	0.37±0.02	0.38±0.02	0.36±0.02	0.35±0.01	0.31±0.03
C20:2	n.d.	n.d.	n.d.	n.d.	0.07±0.12
C20:3	3.6±0.03	4.24±0.12	2.99±0.17	3.31±0.46	3.65±0.39
C20:4	n.d.	n.d.	n.d.	n.d.	n.d.
C20:5	0.88±0.06	0.89±0.06	0.75±0.05	0.45±0.21	0.39±0.14
C22:0	n.d.	n.d.	n.d.	n.d.	n.d.
C22:1	0.34±0.01	0.39±0.01	0.54±0.05	0.72±0.09	0.37±0.1
C22:6	n.d.	n.d.	n.d.	n.d.	n.d.
C24:0	1.13±0.04	1.13±0.03	0.81±0.06	0.62±0.37	0.56±0.01

	122 h				
Fatty acid	Dark	Red680	WhiteSWW	Green510	Blue455
C13:0	0.06±0.11	n.d.	0.12±0.2	0.28±0.09	n.d.
C14:0	4.18±0.08	4.07±0.05	3.76±0.16	3.74±0.14	3.42±0.61
C14:1	0.65±0.06	0.57±0.07	0.42±0.05	0.44±0.06	0.23±0.21
C15:0	1.42±0.04	1.39±0.04	3.43±0.07	4.04±0.5	0.93±0.08
C16:0	9.07±0.2	9.08±0.15	8.66±0.4	9±0.3	14.7±0.21
C16:1	9.75±0.25	8.93±0.21	7.54±0.34	7.48±0.18	6.9±0.45
C17:00	0.35±0	0.43±0.09	0.75±0.03	0.93±0.11	0.81±0.07
C17:1	1.92±0.04	2±0.34	2.91±0.21	3.25±0.32	1.23±0.07
C18:0	0.11±0.1	n.d.	0.14±0.13	0.38±0.3	0.31±0.05
C18:1 (oleat)	2.06±0.03	1.62±0.67	0.85±0.06	0.83±0.08	1.72±0.27
C18:1 (vaccenat)	17.31±0.27	16.75±0.52	15.1±0.54	15.85±0.72	19.58±0.59
C18:2	n.d.	n.d.	n.d.	n.d.	n.d.
C18:3	2.24±0.01	2.26±0.05	2.86±0.12	3.07±0.23	2.17±0.14

C19:00	0.05±0.08	n.d.	n.d.	0.09±0.15	n.d.
C20:0	n.d.	n.d.	n.d.	n.d.	n.d.
C20:1	0.35±0.01	0.31±0.05	0.36±0.02	0.36±0.02	0.38±0.03
C20:2	n.d.	n.d.	n.d.	n.d.	n.d.
C20:3	3.63±0.06	4.14±0.24	3.35±0.15	3.61±0.25	5.28±0.55
C20:4	n.d.	n.d.	n.d.	n.d.	n.d.
C20:5	0.9±0.04	0.88±0.03	0.84±0.08	0.72±0.23	0.89±0.21
C22:0	n.d.	n.d.	n.d.	n.d.	n.d.
C22:1	0.34±0.02	0.44±0.12	0.62±0.03	0.8±0.06	0.43±0.23
C22:6	n.d.	n.d.	n.d.	n.d.	n.d.
C24:0	1.16±0.04	1.13±0.05	0.93±0.07	0.87±0.01	0.44±0.2

Setup 2

	40 h						
	Dark	Red680	WhiteSWW	Green510	Blue470	Blue455	Blue425
C13:0	0.16±0.04	0.11±0.15	0.38±0.02	0.39±0.02	n.d.	n.d.	n.d.
C14:0	4.01±0.11	4.01±0.05	3.49±0.09	3.3±0.03	2.39±0.82	2.8±0.14	3.43±0.14
C14:1	0.62±0.09	0.54±0.02	0.4±0.06	0.33±0	0.25±0.06	0.25±0.06	0.16±0.14
C15:0	1.49±0.07	1.44±0.02	3.51±0.04	3.98±0.14	1.01±0.11	0.76±0.06	0.89±0.01
C16:0	8.5±0.34	8.96±0.47	7.95±0.22	7.74±0.12	11.51±0.25	11.62±0.19	12.06±0.16
C16:1	8.28±0.09	8.31±0.07	7.05±0.1	6.64±0.09	6.12±0.13	5.57±0.14	6.05±0.09
C17:00	0.34±0.01	0.37±0	0.68±0.01	0.77±0.03	0.8±0	0.59±0.01	0.47±0.03
C17:1	1.77±0.03	1.8±0.04	2.85±0.08	3.1±0.06	1.41±0.02	0.93±0.03	1.02±0.04
C18:0	0.13±0.01	0.06±0.09	0.05±0.08	0.13±0.02	0.24±0.02	0.25±0.01	0.23±0.02
C18:1 (oleat)	3.01±0.12	2.81±0.11	0.93±0.05	0.8±0.02	1.06±0.04	1.47±0.01	1.44±0.07
C18:1 (vaccenat)	14.42±0.45	14.64±0.07	13.85±0.39	13.67±0.17	16.79±0.32	14.95±0.27	14.9±0.17
C18:2	n.d.	n.d.	n.d.	n.d.	0.08±0.14	n.d.	n.d.
C18:3	1.93±0.09	1.94±0.02	2.38±0.12	2.48±0.08	1.29±0.33	1.48±0.16	1.81±0.03
C19:00	0.04±0.07	0.07±0.1	n.d.	n.d.	n.d.	n.d.	n.d.
C20:0	n.d.	n.d.	0.21±0.01	0.27±0.02	0.07±0.12	n.d.	n.d.
C20:1	0.27±0.01	0.28±0	0.31±0.02	0.33±0.01	0.23±0.01	0.24±0.01	0.29±0.02
C20:2	n.d.	n.d.	n.d.	n.d.	n.d.	n.d.	n.d.
C20:3	2.9±0.06	3.38±0.05	2.58±0.07	2.6±0.06	3.06±0.03	3.51±0.1	4.16±0.06
C20:4	n.d.	n.d.	n.d.	0.09±0.08	n.d.	n.d.	n.d.
C20:5	0.83±0.04	0.76±0.06	0.69±0.06	0.68±0.05	0.2±0.04	0.22±0.07	0.55±0.07
C22:0	n.d.	n.d.	n.d.	n.d.	n.d.	n.d.	n.d.
C22:1	0.25±0	0.32±0.02	0.37±0.01	0.47±0.02	0.44±0.06	0.38±0.02	0.37±0.01
C22:6	n.d.	n.d.	0.07±0.11	n.d.	n.d.	n.d.	n.d.
C24:0	1.02±0.05	1.14±0.1	0.84±0.05	0.82±0.03	0.58±0.14	0.62±0.01	0.78±0.06

	94 h						
	Dark	Red680	WhiteSWW	Green510	Blue470	Blue455	Blue425
C13:0	0.06±0.11	0.08±0.11	0.26±0.17	0.32±0.17	n.d.	0.07±0.06	0.13±0.03
C14:0	3.85±0.12	3.52±0.31	3.17±0.66	3.22±0.24	2.53±0.45	3.22±0.04	3.72±0.13
C14:1	0.51±0.21	0.56±0.18	0.4±0.05	0.4±0.03	0.33±0.03	0.34±0.02	0.42±0.04
C15:0	1.46±0.03	1.42±0.08	3.63±0.09	4.04±0.12	1.09±0.05	0.92±0.1	1.07±0.14
C16:0	8.61±0.01	8.66±0	8.66±0.03	8.29±0.13	12.24±0.31	13.8±0.17	13.78±0.45

C16:1	9.58±0.07	9.3±0.1	8.28±0.18	7.55±0.04	6.23±0.19	6.24±0.16	6.38±0.15
C17:00	0.36±0.02	0.37±0.01	0.73±0.02	0.79±0.02	0.89±0.02	0.68±0.01	0.54±0.02
C17:1	1.77±0	1.74±0.02	3.21±0.13	3.35±0.03	1.68±0.06	1.15±0.03	1.23±0.04
C18:0	0.04±0.07	0.13±0	0.13±0.01	0.13±0.01	0.23±0	0.27±0.03	0.23±0.05
C18:1 (oleat)	3.27±0.13	2.93±0.15	1.11±0.05	0.83±0.04	0.91±0.1	1.57±0.05	1.4±0.02
C18:1 (vaccenat)	15.37±0.08	15.09±0.08	15.99±0.48	15.1±0.25	18.09±0.62	17.93±0.26	17.33±0.43
C18:2	n.d.	n.d.	0.34±0.59	n.d.	n.d.	n.d.	n.d.
C18:3	1.99±0.12	2.14±0.05	2.67±0.28	2.71±0.06	1.02±0.1	1.77±0.14	2.24±0.06
C19:00	0.14±0.02	0.14±0	0.14±0.01	0.09±0.08	n.d.	0.03±0.05	n.d.
C20:0	n.d.	n.d.	0.09±0.08	0.25±0.04	n.d.	n.d.	n.d.
C20:1	0.28±0.02	0.29±0.01	0.35±0.01	0.32±0.02	0.23±0	0.27±0.01	0.36±0.01
C20:2	n.d.	n.d.	0.2±0.34	n.d.	n.d.	n.d.	n.d.
C20:3	3.2±0.03	3.52±0.1	3.1±0.03	2.88±0.13	3.3±0.14	4.32±0.06	4.87±0.14
C20:4	n.d.	n.d.	n.d.	n.d.	n.d.	n.d.	n.d.
C20:5	0.95±0.02	0.87±0.04	0.67±0.17	0.76±0.07	0.29±0.15	0.37±0.16	0.13±0.04
C22:0	n.d.	n.d.	n.d.	n.d.	0.04±0.07	0.08±0.07	n.d.
C22:1	0.28±0.02	0.33±0.01	0.49±0.03	0.53±0.04	0.27±0.24	0.37±0.22	0.5±0.04
C22:6	n.d.	n.d.	n.d.	n.d.	n.d.	n.d.	n.d.
C24:0	1.24±0.05	1.18±0.01	1.18±0.23	0.93±0.04	0.25±0.18	0.19±0.02	0.25±0.02

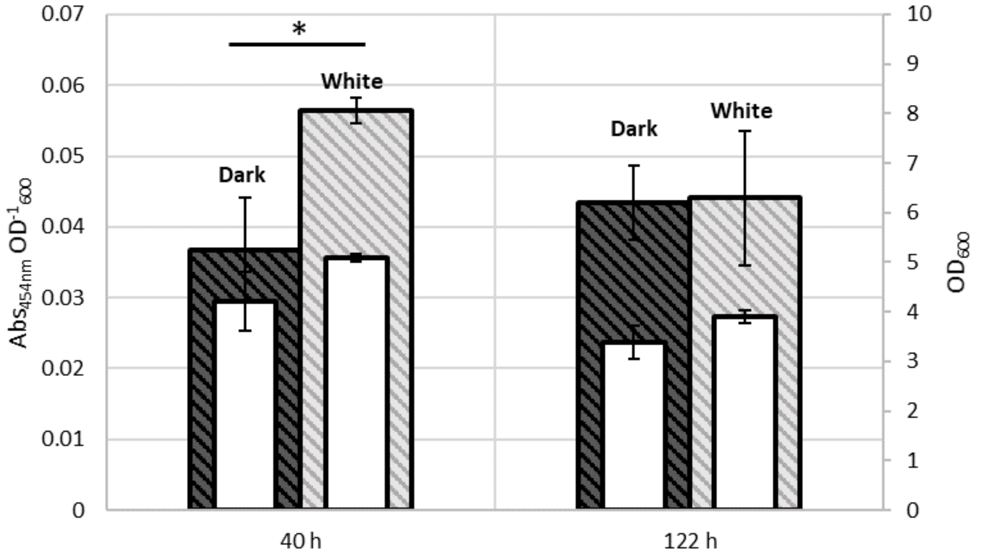

Figure S3. Carotenoid accumulation of proteomic samples normalized on the OD_{600nm} of *R. erythropolis* grown under white light LEDs as well as dark conditions as control, * $p < 0.05$ (n=3). Absorbance measured at 454 nm. (a) OD_{600nm} and carotenoid level after 40 h (b) OD_{600nm} and carotenoid level after 122 h.

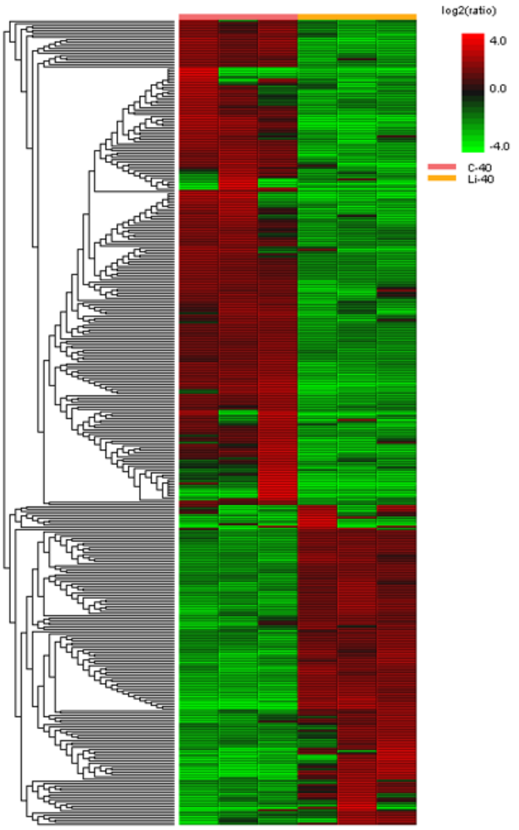

(**a**)

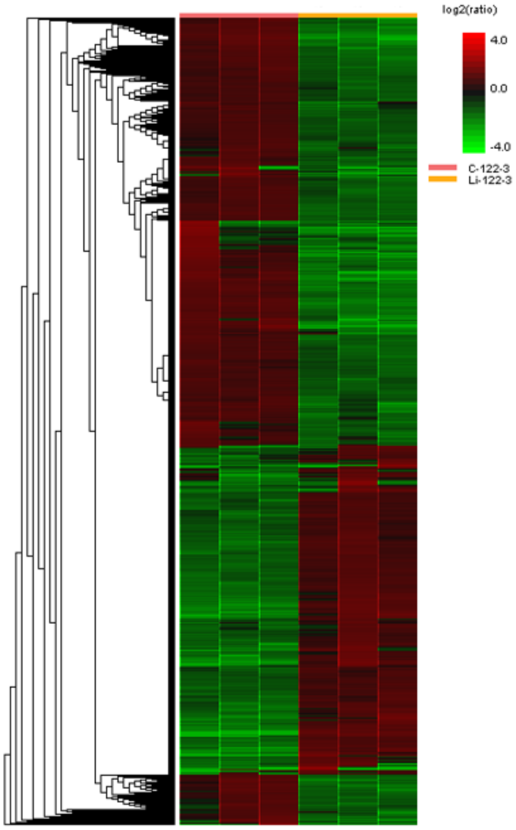

(**b**)

Figure S4. Heat map of the proteins quantified between samples grown under LED light compared to dark conditions as control with significantly different abundance (Significance and fold change ≥ 2). All downregulated proteins are marked in green while upregulated proteins are marked in red. (a) Heat map after 40 h. (b) Heat map after 122 h. Figure compiled by PEAKS Studio Xpro. High resolution figures are provided as separate files (Figure S4a.png and Figure S4b.png).
