# Supplementary figures and images for: Effects of Light on Growth and Metabolism of Rhodococcus erythropolis
Source: Microorganisms. 2022 Aug 20;10(8):1680. doi: 10.3390/microorganisms10081680 (PMC9416670; doi:10.3390/microorganisms10081680)

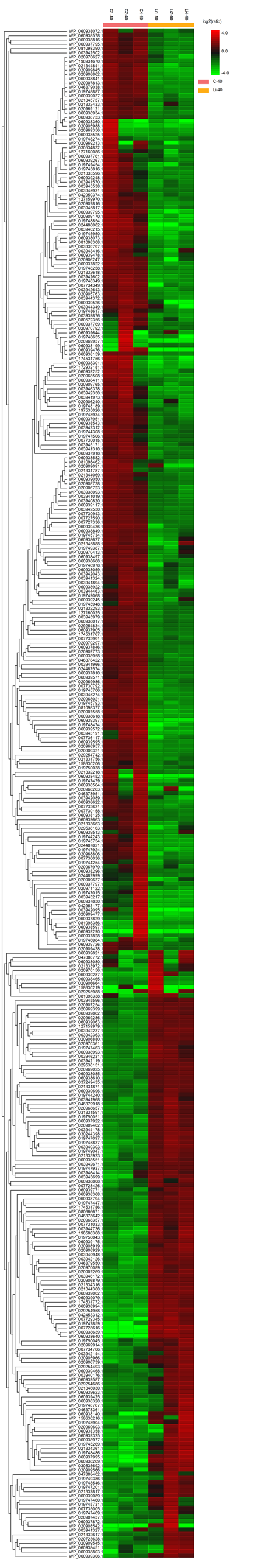

Supplement: Supplementary file 1 [file microorganisms-10-01680-s001.zip › Figure S4a.png]
